# Hierarchical Magnetic Network Constructed by CoFe Nanoparticles Suspended Within “Tubes on Rods” Matrix Toward Enhanced Microwave Absorption

**DOI:** 10.1007/s40820-020-00572-5

**Published:** 2021-01-04

**Authors:** Chunyang Xu, Lei Wang, Xiao Li, Xiang Qian, Zhengchen Wu, Wenbin You, Ke Pei, Gang Qin, Qingwen Zeng, Ziqi Yang, Chen Jin, Renchao Che

**Affiliations:** Laboratory of Advanced Materials, Department of Materials Science and Collaborative Innovation Center of Chemistry for Energy Materials (iChem), Fudan University, Shanghai, 200438 People’s Republic of China

**Keywords:** Hierarchical core–shell MOF-based composites, CoFe nanoparticles, Magnetic network, Microwave absorption

## Abstract

Hierarchical magnetic-dielectric composites are promising functional materials with prospective applications in microwave absorption (MA) field. Herein, a three-dimension hierarchical “nanotubes on microrods,” core–shell magnetic metal–carbon composite is rationally constructed for the first time via a fast metal–organic frameworks-based ligand exchange strategy followed by a carbonization treatment with melamine. Abundant magnetic CoFe nanoparticles are embedded within one-dimensional graphitized carbon/carbon nanotubes supported on micro-scale Mo_2_N rod (Mo_2_N@CoFe@C/CNT), constructing a special multi-dimension hierarchical MA material. Ligand exchange reaction is found to determine the formation of hierarchical magnetic-dielectric composite, which is assembled by dielectric Mo_2_N as core and spatially dispersed CoFe nanoparticles within C/CNTs as shell. Mo_2_N@CoFe@C/CNT composites exhibit superior MA performance with maximum reflection loss of − 53.5 dB at 2 mm thickness and show a broad effective absorption bandwidth of 5.0 GHz. The Mo_2_N@CoFe@C/CNT composites hold the following advantages: (1) hierarchical core–shell structure offers plentiful of heterojunction interfaces and triggers interfacial polarization, (2) unique electronic migration/hop paths in the graphitized C/CNTs and Mo_2_N rod facilitate conductive loss, (3) highly dispersed magnetic CoFe nanoparticles within “tubes on rods” matrix build multi-scale magnetic coupling network and reinforce magnetic response capability, confirmed by the off-axis electron holography.

**Electronic supplementary material:**

The online version of this article (10.1007/s40820-020-00572-5) contains supplementary material, which is available to authorized users.

## Highlights

- Three-dimension hierarchical core–shell Mo_2_N@CoFe@C/CNT composites were successfully constructed via a fast MOF-based ligand exchange strategy.
- Abundant magnetic CoFe nanoparticles suspended within “nanotubes on microrods” matrix exhibited strong magnetic loss capability, confirmed by off-axis electron holography.
- Hierarchical Mo_2_N@CoFe@C/CNT composites displayed remarkable microwave absorption value of − 53.5 dB.

**Electronic supplementary material:**

The online version of this article (10.1007/s40820-020-00572-5) contains supplementary material, which is available to authorized users.

## Graphical abstract

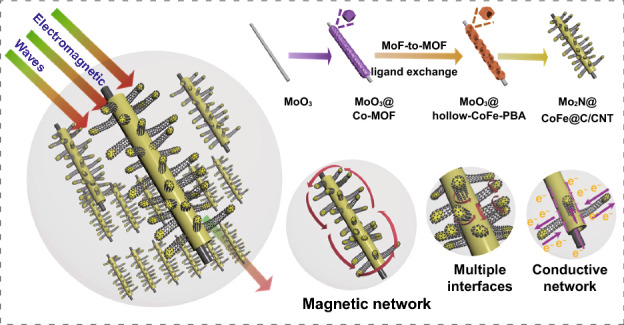

## Introduction

Coming into the fifth-generation (5G) wireless communication systems, the increasing usage of diverse electronic productions has caused severe electromagnetic radiation pollution, which results in an urgent pursuit for high-performance microwave absorption (MA) materials [1–9]. Magnetic materials, including metals (Co, Ni, Fe) and metallic alloys (FeCo, NiCo, etc.), are generally used as microwave absorbents due to strong magnetic loss ability [10–18]. However, practical applications of magnetic materials suffer from their inherent drawbacks: undesirable chemical stability, severe aggregation and inferior impedance matching [19–21]. To tackle these obstacles, two typical strategies have been commonly employed to shape MA properties. One is to decorate magnetic component with carbon materials to develop magnetic-dielectric system and thereby boost the MA performance by enhancing dielectric loss and improving impedance matching [22–31]. For example, Cao et al. designed Fe@NCNTs composite and showed MA performance of − 30.43 dB [32]. Shui et al. prepared CoFe/carbon fiber composite with high MA properties [13]. Tong et al. designed Co/C/Fe/C composite which exhibited significantly improved MA abilities [11]. The other is to construct hierarchical-structured materials with well-designed nano-units, thus achieving high dispersion of magnetic particles and producing heterogeneous interface in multicomponent materials [33–37]. Among various hierarchical structures, the core–shell structures have attracted growing attention in the MA field [38–40] such as Co@C microspheres [10], Fe_3_O_4_/C [41], Co@CoO [42], Co_20_Ni_80_@TiO_2_ core–shell structure [43]. The delicately designed core–shell composites can satisfy magnetic and dielectric loss simultaneously resulting from synergistic effects of different components within both core and shell [44, 45]. Besides, large interspace and heterogeneous interface created by core–shell structure can further enhance polarization loss and strengthen multi-reflection process [37, 44, 46]. Particularly, hierarchical 1D units assembled core–shell composites exhibit remarkable performance in MA application [14, 47, 48]. For example, Che et al. designed hierarchically tubular C/Co composite with abundant 1D nanotubes and achieved highly uniform distribution of Co nanoparticles and outstanding MA performance [49]. Therefore, it is highly desirable to develop a facile and effective preparation strategy to construct magnetic metal–carbon composites with hierarchical core–shell structure.

Metal–organic frameworks (MOFs), with diverse microstructure and adjustable composition, have been widely utilized to construct various hierarchical composites [50–54]. MOF-derived materials demonstrate inherent advantages of abundant metal/carbon components, which endows them with great potential in MA application [40, 55–60]. For example, Ji et al. developed MOF-derived one-dimensional sponge-like metallic Co and Co/C composites with strong magnetic loss [61]. Du et al. presented a MOFs-derived method to construct hollow Co/C microspheres as microwave absorbents [62]. Zhao et al. prepared hierarchical Fe–Co/N‑doped carbon/rGO composites derived from Fe-doped Co-MOF [63]. However, direct transforming MOFs into microwave absorbents leads to a much lower ratio of metal nanoparticles and poor graphitization degree of carbon or CNTs, which is unfavorable to the attenuation of microwave. To tackle these problems, the MOF precursor can be further extended by transforming one kind of MOF into another via ion exchange reactions or ligand exchange reactions, introducing more magnetic metals and carbon components. For example, Hu et al. constructed hierarchical bimetallic Co_2_[Fe(CN)_6_] hollow structure from a Co-MOF through ion exchange reactions [64]. This MOF-to-MOF strategy inspires us to construct bimetallic MOF-derived carbon-based absorbents with favorable hierarchical structure, which has rarely been reported in MA field.

Recently, transition metal molybdenum-based materials, such as MoO_2_, Mo_2_C, MoS_2_ and Mo_2_N, have emerged as effective candidates in the field of electrocatalysis, lithium batteries and supercapacitors due to its low cost, high conductivity and chemical stability [65–72]. Such superior properties also make molybdenum compounds promising microwave absorbents. For example, owing to metallic-like conductivity of MoO_2_ materials, Huang et al. constructed C@MoO_2_/G composites for efficient MA [73]. Du et al. fabricated ternary Mo_2_C/Co/C composites for MA [74] and Jin et al. prepared MoS_2_-NS with high dielectric properties and MA performances [75]. However, the work of employing Mo_2_N as microwave absorbent has not been studied so far, although Mo_2_N materials exhibit satisfied electrical conductivity displaying excellent performance in electrocatalysis and supercapacitors [66, 69]. Therefore, compositing molybdenum compounds into metal–carbon absorbents with designed hierarchical structure is expected to achieve first-rate MA performance.

Herein, for the first time, a 3D hierarchical “nanotubes on microrods” core–shell composite of magnetic CoFe nanoparticles suspended within one-dimensional graphitized C/CNTs supported on Mo_2_N rod (Mo_2_N@CoFe@C/CNT) is successfully achieved through a fast MOF-based ligand exchange strategy. The intermediate product of MoO_3_@hollow-CoFe-PBA composite plays an important role in not only providing Fe source for the growth of CoFe alloy and C/CNTs but also constructing hierarchical core–shell structure in final composite, thus achieving highly dispersive distribution of magnetic particles. The unique Mo_2_N@CoFe@C/CNT composite holds the dielectric Mo_2_N as core and magnetic CoFe nanoparticles embedded C/CNTs as shell. Such 3D hierarchical magnetic network assembled by CoFe nanoparticles suspended within “tubes on rods” matrix demonstrates strong magnetic loss capability, which can be verified by off-axis electron holography. Besides, numerous Mo_2_N rods and graphitized CNTs in the composite constitute dual conductive network to facilitate conductive loss. Moreover, large interfaces in hierarchical core–shell structure can trigger intensive polarization loss. Our hierarchical Mo_2_N@CoFe@C/CNT composite demonstrates superior MA performance with maximum reflection loss value of − 53.5 dB at the thickness of only 2 mm thickness and the effective absorption bandwidth can reach 5.0 GHz. Therefore, the presented fast MOF-based ligand exchange strategy provides an effective method to fabricate multicomponent absorbents with well-controlled hierarchical structure for achieving excellent MA properties.

## Experimental Section

### Materials

All chemicals used were of analytical grade and were used directly without further purification. All chemicals were purchased from Sinopharm Chemical Reagent Co., Ltd.

### Synthesis of MoO_3_

In a typical synthesis, 0.5793 g ammonium molybdate tetrahydrate was dissolved in 30 mL of deionized (DI) water; then, 2.5 mL of HNO_3_ was added. The solution was kept stirring for 10 min, then transferred into a Teflon-lined stainless autoclave (50 mL) and kept at 180 °C for 12 h. When the temperature of Teflon-lined stainless autoclave was cooled naturally, the precipitate was collected and washed repeatedly with DI water for at least three times before drying at 70 °C.

### Synthesis of MoO_3_@Co-MOF

First, the solution A was prepared by 50 mg of MoO_3_ and 0.582 g CoNO_3_·6H_2_O were dissolved in 20 mL of methanol. Then solution B was prepared by dispersing 1.3132 g of 2-methylimidazole in 20 mL of methanol. The solution B was added into solution A under stirring and kept stirring for 5 min then aged for 20 min at room temperature. The precipitate was collected and washed with ethanol for at least three times and dried at 70 °C.

### Synthesis of MoO_3_@hollow-CoFe-PBA

40 mg of MoO_3_@Co-MOF was dissolved in 10 mL ethanol to get solution C. 40 mg of K_3_[Fe(CN)_6_] was dissolved in 20 mL DI water and 20 mL ethanol to get solution D. Then solution D was poured into solution C under stirring and kept stirring for 5 min. The precipitate was collected and washed with DI water and dried at 70 °C.

### Synthesis of Mo_2_N@CoFe@C/CNT

In a typical synthesis, 0.1 g of as-prepared MoO_3_@hollow-CoFe-PBA and 0.5 g of melamine were placed separately in a quartz boat where the melamine was placed at upstream side of the furnace. The furnace was heated to 600 °C at a rate of 2 °C min^−1^ for 4 h under a hydrogen/argon atmosphere. Finally, Mo_2_N@CoFe@C/CNT composite was obtained after cooling down to ambient temperature.

### Synthesis of Mo_2_N and Mo_2_N@Co/CNT

For comparison, Mo_2_N and Mo_2_N@Co/CNT were synthesized by calcining the MoO_3_ and MoO_3_@Co-MOF with melamine, respectively.

### Microwave Absorption Measurements

The measured samples were first prepared by adding the absorbents (20 wt%) into molten paraffin and uniformly mixing them, followed by modeling into a coaxial ring with the outer diameter of 7.0 mm and inner diameter of 3.0 mm. Electromagnetic parameters (complex permittivity and complex permeability) were measured by a N5230C vector network analyzer over the range of 2–18 GHz. The reflection loss values were calculated based on the transmission line theory:

1 $$ Z_{\text{in}} = \sqrt {\mu_{r} /\varepsilon_{r} \tanh \left| { - j(2\pi fd/c)\sqrt {\varepsilon_{r} \mu_{r} } } \right|} $$

2 $$ {\text{RL}}({\text{dB}}) = - 20\log \left| {Z_{\text{in}} - 1/Z_{\text{in}} + 1} \right| $$

where *ε*_*r*_ and *µ*_*r*_ are the complex permittivity (*ε*_*r*_=* ε′ *−* jε′′*) and permeability (*µ*_*r*_=* µ′ *−* jµ′′*), respectively, *f* is the frequency of microwave, *c* is the velocity of light, *d* is the thickness, and *Z*_in_ is the normalized input impedance of the sample.

### Characterizations

The crystalline phase and purity of the products was analyzed by powder X-ray diffraction (XRD, Bruker, D8-Advance X-ray diffractometer, Germany) using Ni-filtered Cu Ka radiation. The morphology and structure of the products were examined by a field-emission scanning electron microscopy (SEM) on a Hitachi S-4800 with an accelerating voltage of 5 kV and a field-emission transmission electron microscope (TEM, JEOL, JEM-2100F, 200 kV). The Raman spectra were acquired with a Renishaw Invia spectrometer using a 514 nm laser excitation. X-ray photoelectron spectroscopy (XPS) spectra were obtained on an ESCALab MKII X-ray photoelectron spectrometer using Al Kα X-ray as the excitation source. The hysteresis loops were performed with a superconducting quantum interference device (MPMS(SQUID) VSM) magnetometer (Quantum Design Company).

## Results and Discussion

### Fabrication and Characterization of Mo_2_N@CoFe@C/CNT Composites

**Fig. 1:**
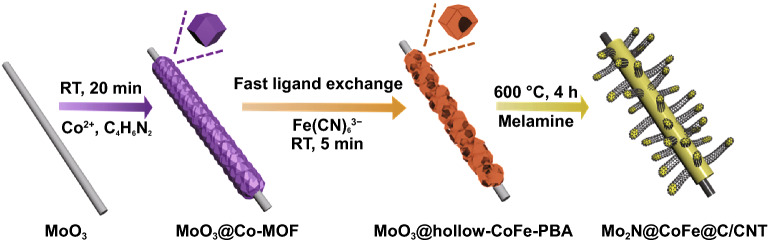
Schematic process of the fast MOF-based ligand exchange strategy for construction of 3D hierarchical Mo_2_N@CoFe@C/CNT composites

The synthesis of the hierarchical Mo_2_N@CoFe@C/CNT core–shell structure is illustrated in Fig. 1. First, the Co-MOF is uniformly grown on MoO_3_ rod to form MoO_3_@Co-MOF structure. Second, through a fast ligand exchange reaction with K_3_[Fe(CN)_6_] in 5 min at room temperature, MoO_3_@Co-MOF structure is in situ converted into MoO_3_@hollow-CoFe-PBA core–shell composite. Followed by the carbonization of MoO_3_@hollow-CoFe-PBA with melamine, the inner MoO_3_ is transformed into Mo_2_N rod and the outer hollow-CoFe-PBA turn into the CoFe@C/CNTs architecture, where thermally reduced CoFe nanoparticles could catalyze the growth of graphitic carbon and CNTs with melamine as carbon source. Finally, the hierarchical Mo_2_N@CoFe@C/CNT composite with “tubes on rods” structure is successfully obtained. Moreover, through fast ligand exchange reaction, the intermediate product of MoO_3_@hollow-CoFe-PBA core–shell structure plays a critical role in the formation of hierarchical Mo_2_N@CoFe@C/CNT composite, which will be explained in the following discussion.

**Fig. 2:**
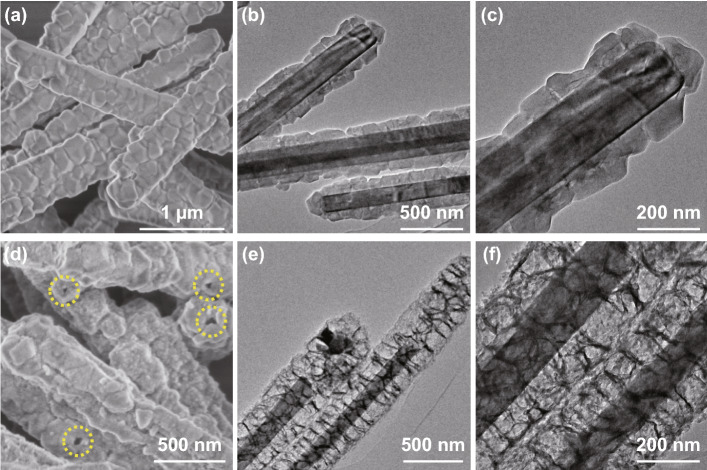
**a** SEM, **b**, **c** TEM images of MoO_3_@Co-MOF, **d** SEM, **e**, **f** TEM images of MoO_3_@hollow-CoFe-PBA composites

As displayed in Fig. S1, as-prepared uniform MoO_3_ rods demonstrate smooth surface and high phase purity. Then MoO_3_ rods are covered with Co-MOF to form MoO_3_@Co-MOF structure. The SEM images reveal that the surface of MoO_3_ rods becomes rough (Fig. 2a). The core of MoO_3_ and shell of Co-MOF can be clearly observed in TEM images (Fig. 2b, c). And both diffraction peaks of MoO_3_ and Co-MOF are well detected in XRD pattern (Fig. S2), indicating that Co-MOF is successfully grown on the MoO_3_ rods. To construct hollow CoFe-PBA on the MoO_3_ rods, the MoO_3_@Co-MOF samples are kept in K_3_[Fe(CN)_6_] solution and stirred for just 5 min at room temperature to allow the ligand exchange reaction to prepare MoO_3_@hollow-CoFe-PBA structure. Firstly, the MoO_3_@Co-MOF will slowly decompose in water/ethanol to release Co^2+^ ions. Then the [Fe(CN)_6_]^3−^ ions are injected into the reaction solution. The released Co^2+^ ions can interact with [Fe(CN)_6_]^3−^ ions to generate CoFe-PBA shell around the framework of the precursors (Co-MOF). Finally, the solid Co-MOF shell is completely converted into hollow CoFe-PBA, and MoO_3_@hollow-CoFe-PBA core–shell composites are obtained. As displayed in Fig. 2d, the rather rough CoFe-PBA is grown on the MoO_3_ rods and some holes can be seen on the surface (as displayed in the yellow circles of Fig. 2d). Such unique shell of hollow CoFe-PBA can be further confirmed by TEM images. In Fig. 2e, f, the as-prepared MoO_3_@hollow-CoFe-PBA structure is consisted of the nanocage-assembled CoFe-PBA shell and the MoO_3_ core. XRD result also demonstrates that the sample is composed of MoO_3_ and Co_2_[Fe(CN)_6_] (Fig. S3) [64]. Such core–shell of MoO_3_@hollow-CoFe-PBA composite plays a significant role not only in providing the Fe source for the growth of CoFe alloys and CNTs but also in constructing the core–shell structure in the final multicomponent products. Subsequently, the MoO_3_@hollow-CoFe-PBA composite is converted into Mo_2_N@CoFe@C/CNT core–shell structure through the carbonization with melamine. For comparison, Mo_2_N rod and Mo_2_N@Co/CNT samples are also synthesized by calcining the MoO_3_ and MoO_3_@Co-MOF composite with melamine, respectively.

**Fig. 3:**
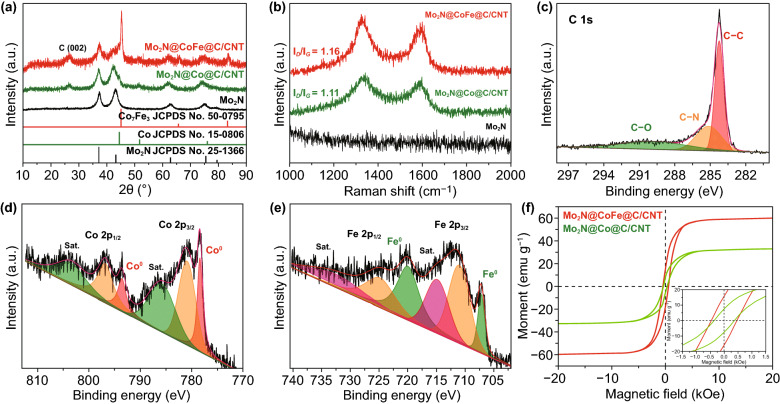
**a** XRD patterns, **b** Raman spectra of as-prepared Mo_2_N, Mo_2_N@Co/CNT and Mo_2_N@CoFe@C/CNT. High resolution XPS spectra of **c** C 1*s*, **d** Co 2*p*, and **e** Fe 2*p* for Mo_2_N@CoFe@C/CNT composite. **f** hysteresis loops of Mo_2_N@Co/CNT and Mo_2_N@CoFe@C/CNT composites

The chemical compositions of Mo_2_N rod, Mo_2_N@Co/CNT, and Mo_2_N@CoFe@C/CNT composites are measured by XRD, Raman and XPS techniques. As displayed in Fig. 3a, the diffraction peaks of Mo_2_N rods are in accordance with reflections of molybdenum nitride (Mo_2_N, JCPDS No. 25-1366) while the Mo_2_N@Co/CNT samples exhibit diffraction peaks of both Mo_2_N and cubic cobalt (JPCDS No. 15-0806). In the XRD pattern of Mo_2_N@CoFe@C/CNT composites, apart from characteristic peaks of Mo_2_N, a diffraction peak at 26.1^o^ can be observed clearly, attributing to the (002) plane of the graphitic carbon. Other peaks at around 45.2^o^, 65.8^o^, and 83.3^o^ match well with diffractions of the cubic cobalt iron (JPCDS No. 50-0795). Above-mentioned XRD results demonstrate that the Mo_2_N@CoFe@C/CNT composite is consisted of Mo_2_N, CoFe alloy and graphitic carbon. To reveal the graphitic feature and structural defects of as-prepared samples, Raman spectra are conducted. In Fig. 3b, the Mo_2_N@CoFe@C/CNT composite exhibits the highest *I*_D_/*I*_G_ value of 1.16 because a great number of defects are produced in such core–shell structure. The value of *I*_D_/*I*_G_ is increased with more CNTs catalyzed by the CoFe alloy compared with less graphitic carbon by single metal Co in Mo_2_N@Co/CNT sample, which could promote the electronic transportation ability. Chemical valence states of Mo_2_N@CoFe@C/CNT are examined via XPS technique. In Fig. 3c, three peaks of C 1*s* spectrum correspond to the C–C (284.28 eV), C–N (285.16 eV) and C–O (189.73 eV) [76]. In the Co 2*p* spectrum, peaks at 778.32 and 793.44 eV are ascribed to Co^0^ in Co 2*p*_3/2_ and Co 2*p*_1/2_ and peaks at 780.82 and 796.67 eV belong to Co^2+^ species. In Fig. 3e, the Fe 2*p* spectrum can be decomposed into two peaks of 707.22 eV for Fe^0^ 2*p*_3/2_ and 719.97 eV for Fe^0^ 2*p*_1/2_ and other two peaks of 711.06 and 724.85 eV for Fe^2+^ 2*p*_3/2_ and 2*p*_1/2_, respectively [63, 77–79]. The bimetal CoFe with multiple valency in Mo_2_N@CoFe@C/CNT sample could result in higher saturation magnetization. As shown in Fig. 3f, the saturation magnetization (*M*s) value of Mo_2_N@CoFe@C/CNT is 59.6 emu g^−1^, which is higher than that of Mo_2_N@Co/CNT sample. And the coercivity value is 449.6 Oe for Mo_2_N@CoFe@C/CNT composite. Such high saturation magnetization and low coercivity of Mo_2_N@CoFe@C/CNT hierarchical structure could boost magnetic storage and reinforce magnetic loss, further promoting MA performance [57, 80].

**Fig. 4:**
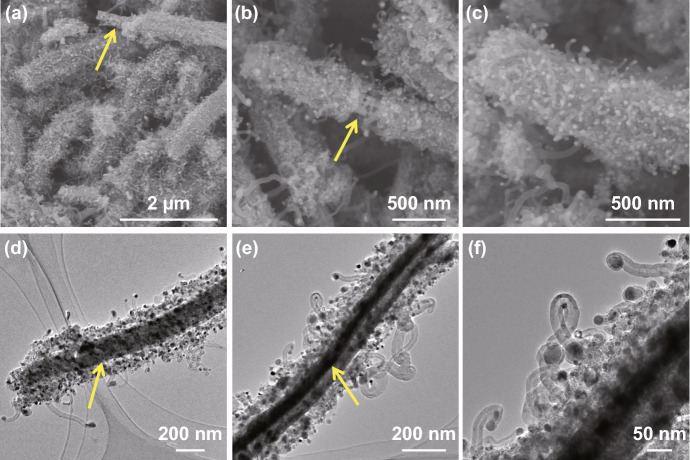
**a–c** SEM, **d–f** TEM images of Mo_2_N@CoFe@C/CNT composites

**Fig. 5:**
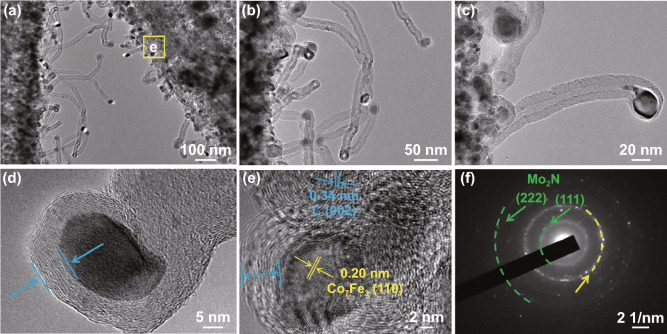
**a–c** The magnified TEM, **d–e** HRTEM images and **f** corresponding selected area electron diffraction of Mo_2_N@CoFe@C/CNT composites

The morphology and structure of Mo_2_N rod, Mo_2_N@Co/CNT and Mo_2_N@CoFe@C/CNT core–shell composites are further performed with SEM and TEM images. As displayed in Fig. 4, a large number of CNTs are produced and deposited on the core of Mo_2_N rod which can be clearly observed in Fig. 4a–c with yellow arrows. In the following TEM images, the rod-like core is seen and wrapped by outer shell of numerous CNTs. Particularly, the obvious void exists between the shell and core (Fig. 4d–f) and the CNTs are not directly grown on the Mo_2_N rod but supported by the shell of CoFe alloy embedded graphitic carbon layers. Such uniquely hierarchical Mo_2_N@CoFe@C/CNT core–shell structure is reported for the first time and can be further confirmed by the magnified TEM and HRTEM images. Abundant CNTs can be seen and on the top of each CNT is encapsulated metal nanoparticles, which are wrapped by numbers of graphitic carbon layers (Fig. 5a–d). In Fig. 5e, the HRTEM image obtained from the shell of such Mo_2_N@CoFe@C/CNT structure (as marked in Fig. 5a with yellow square) demonstrates that the interplanar spacing of 0.20 nm can correspond to the (110) plane of CoFe alloy and 0.34 nm to the (002) plane of graphitic carbon, which convincingly confirms such unique shell of CoFe nanoparticles embedded graphitic carbon. The corresponding selected area electron diffraction pattern displays a series of diffraction rings which can be well indexed to diffraction planes of crystalline Mo_2_N and CoFe alloy (Fig. 5f). Clearly, based on the above results of morphology and composition, hierarchical Mo_2_N@CoFe@C/CNT “tubes on rods” architecture is successfully synthesized through a fast MOF-based ligand exchange strategy. In the calcinating process of MoO_3_@hollow-CoFe-PBA composite with melamine, the MoO_3_ is converted into the core of Mo_2_N rod and hollow-CoFe-PBA is transformed into the shell of CoFe alloy embedded C/CNTs with thermally reduced CoFe nanoparticles as catalysts and melamine as carbon source. For comparison, Mo_2_N@Co/CNT sample is obtained by directly annealing MoO_3_@Co-MOF composite with melamine. As shown in Fig. S4, the Mo_2_N@Co/CNT composites maintain the rod structure but only few of CNTs are observed on the surface of Mo_2_N rod without the shell of metal-embedded graphitic carbon framework. This evidence suggests that single Co nanoparticles could not effectively catalyze the growth of CNTs. Obviously, MoO_3_@hollow-CoFe-PBA structure constructed by ligand exchange reaction critically determines the formation of CoFe nanoparticles, graphitic C/CNTs and hierarchical core–shell structure. Rod-like Mo_2_N are prepared through annealing MoO_3_ rods with melamine, which displays uniformly smooth rod structure (Fig. S5). Remarkably, as-prepared hierarchical Mo_2_N@CoFe@C/CNT can be considered as both distinct conductive structure and magnetic network, which hold great potential to achieve superior MA ability.

### Electromagnetic Parameters Analysis and Microwave Absorption Ability

Related electromagnetic parameters of as-prepared Mo_2_N@CoFe@C/CNT, Mo_2_N@Co/CNT and Mo_2_N samples are investigated to reveal the impacts of structure and composition on the MA performance. Generally, MA properties are highly determined by the complex permittivity and complex permeability of materials. It is acknowledged that the real parts of complex permittivity (ε′) and complex permeability (μ′) indicate the capability of storing electromagnetic energy, while the imaginary parts (ε″, μ″) imply the ability to loss electromagnetic energy. As shown in Fig. S8, the pure Mo_2_N sample displays real permittivity (ε′) ranging from 12.06 to 10.92, suggesting the Mo_2_N is a better dielectric material. And the ε′ values of Mo_2_N@Co/CNT samples rise obviously from 19.40 to 12.76 due to the introduction of conductive CNTs. When more CoFe alloy embedded CNTs and graphitic carbon layers are introduced, the ε′ values of Mo_2_N@CoFe@C/CNT sample range from 10.2 to 5.6 with the increase in frequency, demonstrating Mo_2_N@CoFe@C/CNT materials gain strong capability of energy storage and high dielectric polarization. And the ε″ values of Mo_2_N@CoFe@C/CNT also remain high from 3.78 to 2.56, which means a powerful dielectric loss ability. This can be ascribed to the hierarchical conductive network and enhanced interfacial polarization resulting from unique core–shell structure of dielectric Mo_2_N and conductive C/CNTs components. To further evaluate the dielectric loss property, the dielectric loss tangent *δ*_*ε*_ (tan *δ*_*ε*_=* ε″/ε′*) was calculated. It is believed that higher tan *δ*_*ε*_ value means more electric energy of incident microwaves would be dissipated. As shown in Fig. S9a, the tan *δ*_*ε*_ values of Mo_2_N@CoFe@C/CNT remain high, which offers the convincing evidence that the design of hierarchically core–shell structure with the combination of dielectric Mo_2_N and graphitic C/CNTs components is an effective way to enhance the dielectric loss capacity. As for the real (*µ*′) and imaginary (*µ*″) parts of permeability, the *µ*′ and *µ*″ values of Mo_2_N remain close to 1 and 0 due to its nonmagnetic property. Compared with Mo_2_N@Co/CNT samples, the *µ*′ and *µ*″ of Mo_2_N@CoFe@C/CNT are higher because of its enhanced magnetic CoFe alloy component and hierarchical 3D magnetic network. Therefore, the Mo_2_N@CoFe@C/CNT material is prone to generate favorable magnetic loss capability. Based on above discussion, as-prepared Mo_2_N@CoFe@C/CNT composite is expected to exhibit superior MA capability originating from its both synergetic strong dielectric dissipation and magnetic loss.

**Fig. 6:**
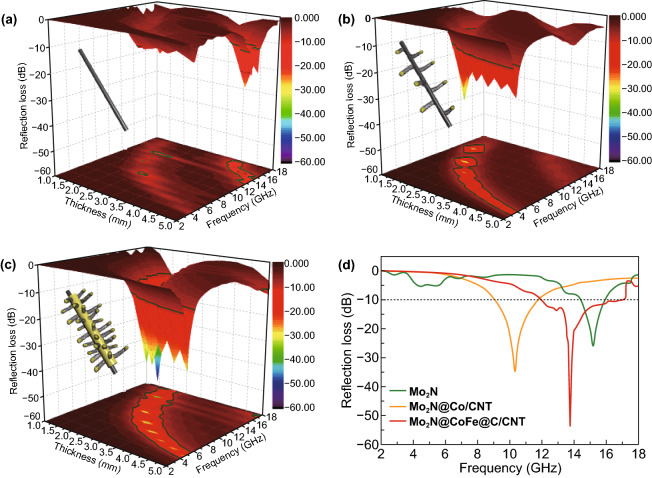
3D plots of reflection loss of **a** Mo_2_N, **b** Mo_2_N@Co/CNT and **c** Mo_2_N@CoFe@C/CNT samples. **d** Reflection loss curves at the same thickness of 2 mm

The MA performance of absorbents is generally evaluated with the maximum reflection loss (*RL*) value and effective absorption bandwidth. Figure 6 displays the 3D plots of *RL* values on different thickness of Mo_2_N, Mo_2_N@Co/CNT and Mo_2_N@CoFe@C/CNT samples. The Mo_2_N rods exhibit good MA performance with the maximum *RL* value of − 25.9 dB at the thickness of 4.5 mm (Fig. 6a) due to its high dielectric property. With the introduction of Co/CNTs components, the Mo_2_N@Co/CNT materials exhibit MA with the maximum *RL* value of − 34.8 dB. Significantly, as displayed in Fig. 6c, the Mo_2_N@CoFe@C/CNT demonstrates the best MA performance with highest maximum *RL* value of − 53.5 dB at the thickness of only 2 mm thickness, and the effective absorption bandwidth can reach 5 GHz (from 12 to 17 GHz). Moreover, while tuning the thickness from 1.5 to 5.0 mm, Mo_2_N@CoFe@C/CNT samples still exhibit impressive MA performance with the maximum *RL* values all less than − 10 dB, revealing its tunable MA ability. These encouraging results demonstrate that as-prepared Mo_2_N@CoFe@C/CNT composites hold excellent MA performance owing to its strong microwave energy absorption, broad effective absorption bandwidth, lower thickness and tunable absorption frequency, which is superior to those reported metal/carbon microwave absorbents (Table S1).

### Analysis of Microwave Absorption Mechanism

**Fig. 7:**
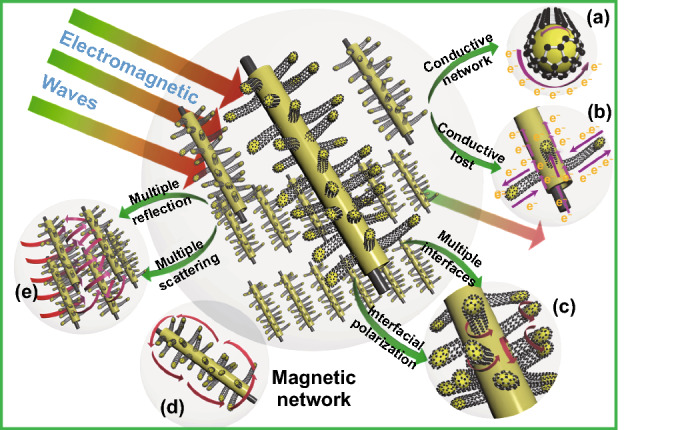
The microwave absorption mechanism in the 3D hierarchical Mo_2_N@CoFe@C/CNT composites

Accordingly, the rational design of 3D hierarchical core–shell structure of Mo_2_N@CoFe@C/CNT absorber and the combination of dielectric Mo_2_N, conductive C/CNTs and magnetic CoFe alloy components contribute to the enhancement of electromagnetic storage and MA performance. Related microwave energy absorption/conversion mechanisms of MA can be illustrated as followed in detail (Fig. 7).

#### Multiple Heterojunction Interfaces and Hierarchical Electronic Transportation Paths Boosted Dielectric Loss

3D assembly Mo_2_N@CoFe@C/CNT composites possess plentiful heterojunction interfaces, which is necessary to the improvement of dielectric storage ability and polarization behaviors. Hierarchical Mo_2_N@CoFe@C/CNT composite is made up of dielectric Mo_2_N, graphitized C/CNTs and magnetic CoFe nanoparticles. In such “tubes on rods” matrix, there are at least three kinds of heterojunction interfaces, including CoFe-CNTs interfaces, graphitized carbon–CNTs interfaces and graphitized carbon–Mo_2_N interfaces (Fig. 7c). Due to differences in electrical conductivity among components, free electrons gather around those contacting interfaces when applied variation of electromagnetic wave. This electronic migration/moment can produce intensive interfacial polarization and relaxation causing the conversion from electromagnetic waves energy into thermal energy. Besides, numerous carbon heteroatoms groups (such as C-N and C-O, Fig. 3c) in Mo_2_N@CoFe@C/CNT could be regarded as active dipole sites. Related dipole polarization can also improve the MA performance. Therefore, Mo_2_N@CoFe@C/CNT composite exhibits higher dielectric polarization ability compared with Mo_2_N@Co/CNT and Mo_2_N materials owing to its multiple interfaces and multicomponent. In addition, both dielectric Mo_2_N rod and graphitized C/CNTs can be also considered as a conductive network. Micro-scale Mo_2_N rod displays high permittivity. When graphitized C/CNTs grow on Mo_2_N rod, numerous electronic transportation routes are formed between C/CNTs and Mo_2_N rod (Fig. 7a, b). This conduction transportation network facilitates enhanced conduction loss capability, which is also favorable for MA performance.

#### Spatial Dispersed CoFe Nanoparticles Built Multi-scale Magnetic Coupling Network

**Fig. 8:**
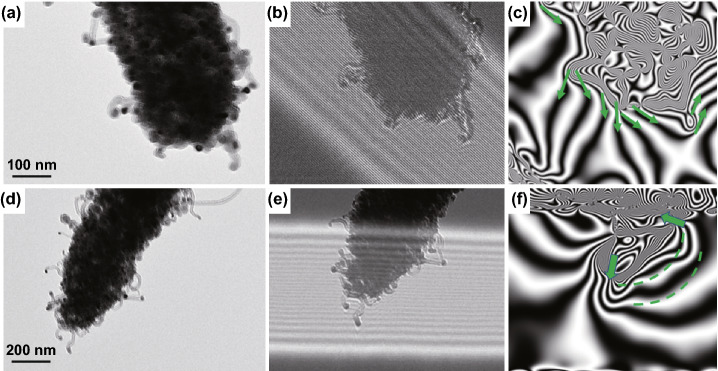
**a**, **d** TEM images and **b–c**, **e–f** corresponding off-axis electron holograms of Mo_2_N@CoFe@C/CNT composites

Spatial dispersed nano-scale CoFe alloy suspended within hierarchical micro-scale Mo_2_N@C/CNTs rod construct a multi-scale magnetic network and could significantly contribute to the boosted magnetic responding capacity (Fig. 7d). Traditionally, magnetic nanoparticles could easily aggregate together due to their magnetic nature. Metal aggregation problem can hardly be avoided in the process of pyrolyzing MOFs directly. Herein, through our ligand exchange strategy, as-synthesized MoO_3_@hollow-CoFe-PBA structure can not only effectively reduce the aggregation of magnetic nanoparticles but also expand spatial magnetic distribution, thereby further increasing the responding scale of magnetic component in the final Mo_2_N@CoFe@C/CNT composite. As-fabricated hierarchical Mo_2_N@C/CNT architecture provides a perfect nano/micro-matrix to support suspended CoFe nanoparticles (Figs. 4 and 5), thus forming a distributed magnetic network and strengthening magnetic permeability. The off-axis electron holography is performed to study the magnetic property of CoFe nanoparticles and related magnetic network in Mo_2_N@CoFe@C/CNT composite. As shown in Fig. 8a–c, the CoFe nanoparticles in the composite can radiate out high-density magnetic lines which could penetrate through the nonmagnetic graphitic C/CNTs and expand magnetic responding regions beyond itself size. Furthermore, the neighbored CoFe nanoparticle suspended within C/CNTs matrix displays magnetic coupling lines which could contribute to integral magnetic network, further strengthen magnetic dissipation capacity (Fig. 8d–f) [49]. Meanwhile, high loading and uniformly distribution of CoFe nanoparticles (Fig. 5) can also enhance the magnetic loss to promote MA performance. Therefore, compared with Mo_2_N@Co/CNT and other magnetic metal/carbon composites reported previously, hierarchical Mo_2_N@CoFe@C/CNT composite can successfully avoid magnetic metal aggregation problem and exhibit remarkable magnetic loss property.

#### Synergic Magnetic-dielectric MA System and Multi-dimension Hierarchical Structure

Hierarchical Mo_2_N@CoFe@C/CNT composites can effectively dissipate the microwave energy via dielectric dissipation and magnetic loss. The assembled composite is constructed by dielectric Mo_2_N as core and spatially dispersed CoFe nanoparticles within C/CNTs as shell and thus demonstrate significantly improved MA performance resulting from both dielectric loss and magnetic loss, compared with single Mo_2_N material or Mo_2_N@Co/CNT composite with few metal nanoparticles. Meanwhile, because of the hierarchical structure and multi-scale size, Mo_2_N@CoFe@C/CNT assembly possess unique multi-reflection and multi-scattering (Fig. 7e). Abundant 1D CNTs, micro-scale Mo_2_N rod and 3D hierarchical core–shell structure could generate effective surface area and spacing effect. When incidence microwave permeates into this 3D architecture, expected large surface areas offer many active sites to produce multiple reflection and scattering. Such repeated reflection and scattering process of incident microwave can successfully attenuate microwave energy. Benefiting from above advantages of hierarchical structure and multi-loss mechanism, as-prepared Mo_2_N@CoFe@C/CNT composites exhibit superior MA performance that surpass those reported metal–carbon microwave absorbents (Table S1).

## Conclusion

In conclusion, as-prepared Mo_2_N@CoFe@C/CNT composites exhibit superior MA performance with maximum reflection loss value of − 53.5 dB at the thickness of only 2 mm thickness and a broad effective absorption bandwidth of 5 GHz. Such 3D hierarchical core–shell structure assembled by nano-scale magnetic CoFe nanoparticles suspended within graphitic C/CNTs supported on micro-scale Mo_2_N rod is rationally constructed via our effective ligand exchange strategy. The dielectric Mo_2_N and C/CNTs components can shape strong conductive loss and hierarchical core–shell structure offers large interfacial area to trigger polarization loss. Moreover, distributed magnetic CoFe nanoparticles embedded in C/CNTs matrix form multi-scale magnetic network and reinforce magnetic response capability, which is verified by the off-axis electron holography. Firmly, the MOF-based ligand exchange strategy in this work can be utilized to construct various hierarchical structure of multicomponent metal–carbon system for enhanced MA performance.

## Electronic supplementary material

Below is the link to the electronic supplementary material.

Supplementary material 1 (PDF 783 kb)
