# Supplementary material for: Hierarchical Magnetic Network Constructed by CoFe Nanoparticles Suspended Within “Tubes on Rods” Matrix Toward Enhanced Microwave Absorption
Source: Nanomicro Lett. 2021 Jan 4;13:47. doi: 10.1007/s40820-020-00572-5 (PMC8187526; doi:10.1007/s40820-020-00572-5)
Supplement: Supplementary file 1 — Supplementary material 1 (PDF 783 kb) [file 40820_2020_572_MOESM1_ESM.pdf]

Supporting Information for

# Hierarchical Magnetic Network Constructed by CoFe Nanoparticles Suspended within "Tubes on Rods" Matrix Toward Enhanced Microwave Absorption

Chunyang Xu[1], Lei Wang[1], Xiao Li[1], Xiang Qian[1], Zhengchen Wu[1], Wenbin You[1], Ke Pei[1], Gang Qin[1], Qingwen Zeng[1], Ziqi Yang[1], Chen Jin[1], Renchao Che[1, *]

[1]Laboratory of Advanced Materials, Department of Materials Science and Collaborative Innovation Center of Chemistry for Energy Materials (iChem), Fudan University, Shanghai 200438, P. R. China

*Corresponding author. E-mail: rcche@fudan.edu.cn (Renchao Che)

## Supplementary Figures and Table

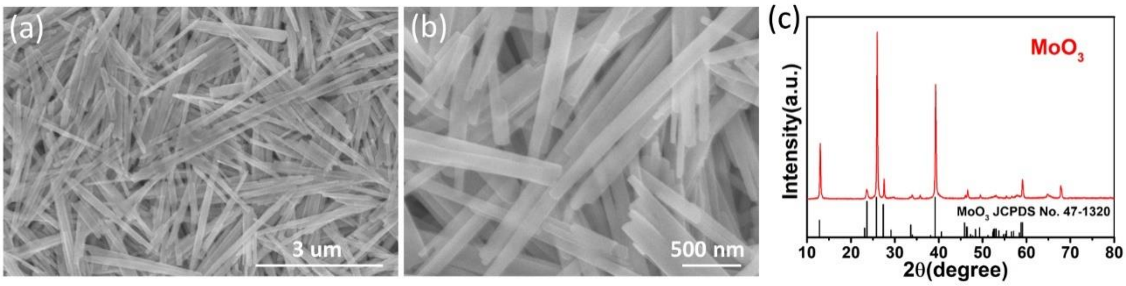


**Fig. S1 a, b** SEM images and **c** XRD patterns of $MoO_3$ samples

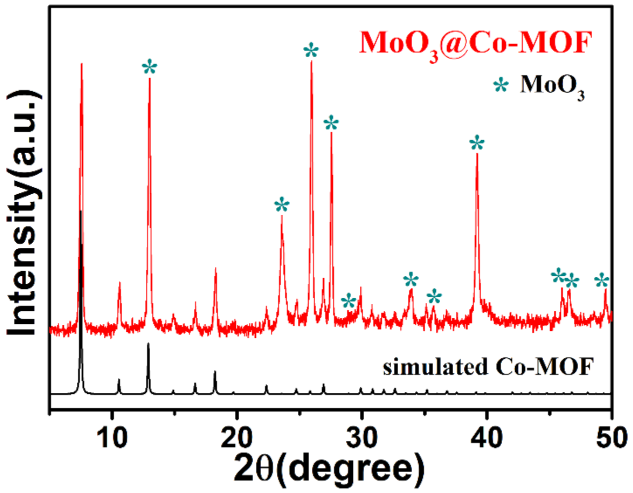


**Fig. S2** XRD patterns of $MoO_3$@Co-MOF

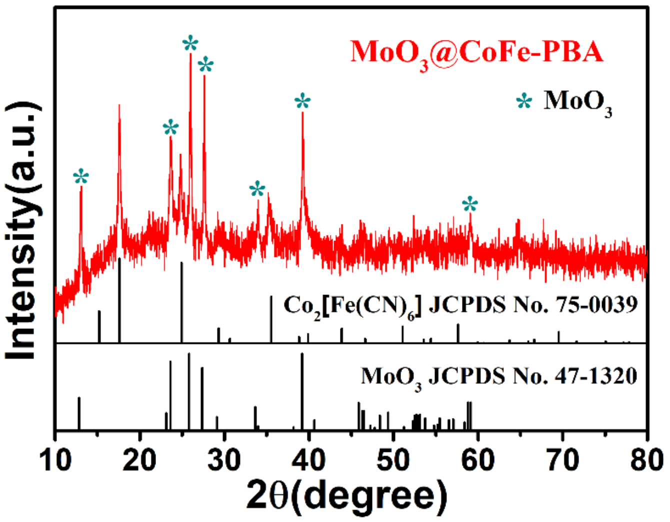


**Fig. S3** XRD patterns of $MoO_3$@hollow-CoFe-PBA samples

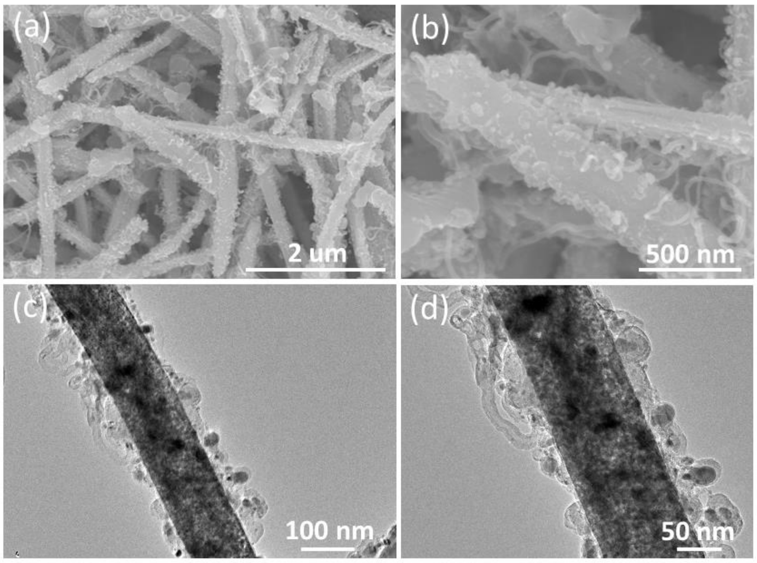


**Fig. S4 a, b** SEM **c, d** TEM images of $Mo_2N$@Co/CNT samples

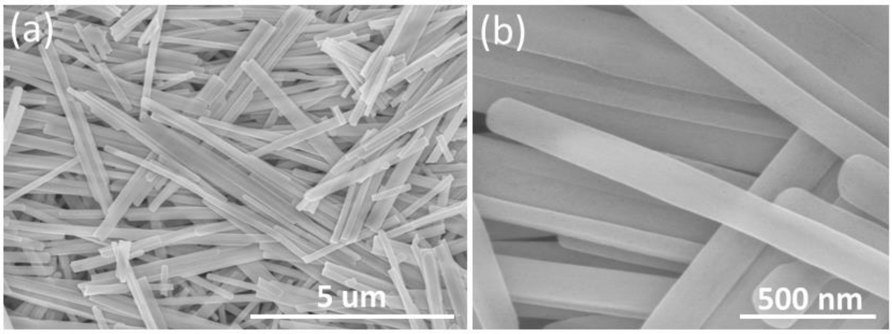


**Fig. S5 a, b** SEM images of $Mo_2N$ samples

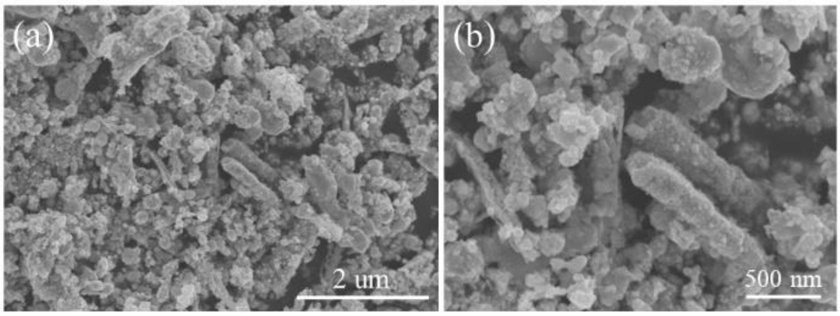


**Fig. S6 a, b** SEM images of samples calcined at 700 °C We have also prepared the sample obtained under 700 °C. As displayed in Fig. S6, $MoO_3$@hollow-CoFe-PBA samples were unstable at 700 °C and decomposed into irregular structure. Even some samples could retain rod-like structure, there were no CNTs on the surface.

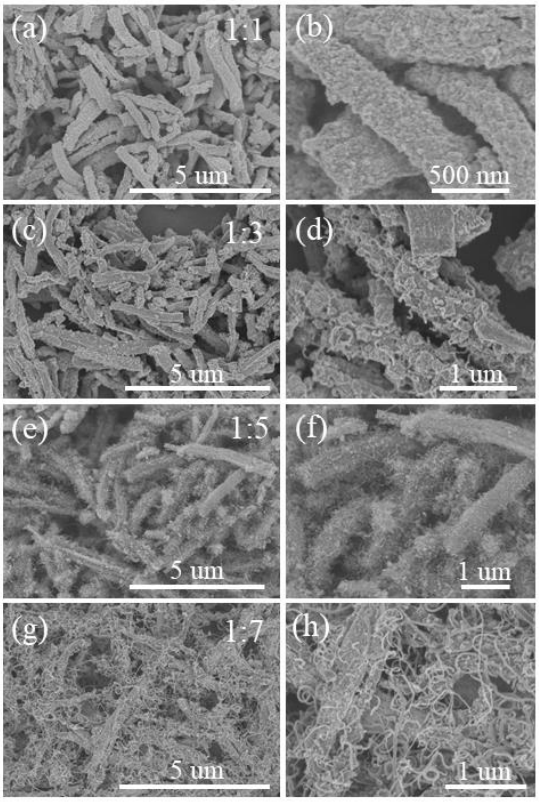


**Fig. S7** SEM images of samples calcined under different weight ratio of $MoO_3$@hollow-CoFe-PBA to melamine: **a-b** 1:1, **c-d** 1:3, **e-f** 1:5, **g-h** 1:7

We have prepared samples under different weight ratio of $MoO_3$@hollow-CoFe-PBA to melamine, including 1:1, 1:3, 1:5, and 1:7. Under the carbonization of $MoO_3$@hollow-CoFe-PBA with the same weight of melamine (1:1), there is no CNTs produced on the $Mo_2N$ rod due to less melamine as carbon sources. Increase the content of melamine (1:3), few CNTs can be seen on the $Mo_2N$ rod. When the weight ratio of $MoO_3$@hollow-CoFe-PBA to melamine is 1:5, a large number of CNTs are produced and deposited on the core of $Mo_2N$ rod, forming $Mo_2N$@CoFe@C/CNT core-shell structure. Increasing the weight ratio to 1:7, too many CNTs are produced and core-shell structure cannot be maintained.

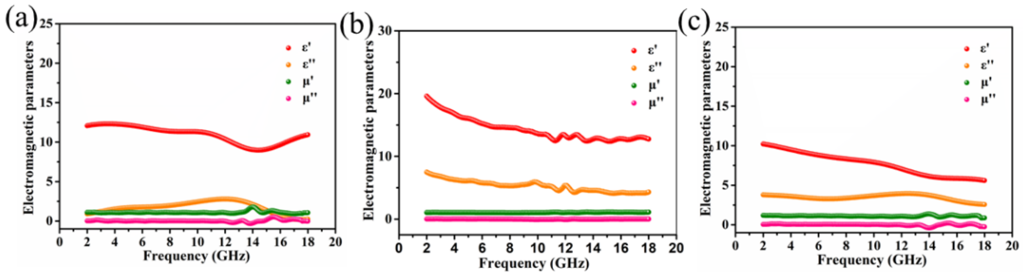


**Fig. S8** Electromagnetic parameters of **a** $Mo_2N$, **b** $Mo_2N$@Co/CNT and **c** $Mo_2N$@CoFe@C/CNT samples

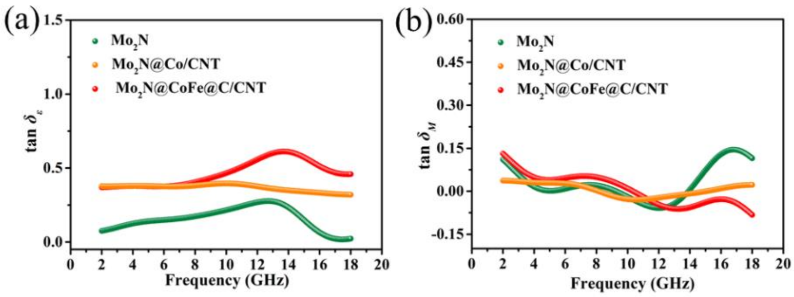


**Fig. S9** **a** Dielectric loss tangent and **b** magnetic loss tangent of $Mo_2N$, $Mo_2N$@Co/CNT, and $Mo_2N$@CoFe@C/CNT samples

**Table S1** Microwave absorption performance of metal/C absorbents and molybdenum-based materials in previous references and this work.

| **Absorbents** | **Thickness (mm)** | **Maximum RL (dB)** | **EAB (GHz)** | **Refs.** |
|---|---|---|---|---|
| FeCo/C/$BaTiO_3$ | 2.0 | -41.7 | 4.2 | [S1] |
| NiFe@C | 2.8 | -51 | 3.97 | [S2] |
| Co/NPC@Void@CI | 2.2 | -49.2 | 6.72 | [S3] |
| MOF (Fe)/PANI | 2.0 | -41.4 | 5.5 | [S4] |
| MWCNT/Fe | 4.3 | -39.0 | 2.7 | [S5] |
| Fe/C | 3.0 | -36.0 | 0.9 | [S6] |
| Fe/C | 2.0 | -22.6 | 5.3 | [S7] |
| Co/N-C NF | 2.0 | -41.7 | 4.2 | [S8] |
| CoZn@NCNTHS/G | 1.5 | -47.3 | 4.0 | [S9] |
| CMT@CNT/Co | 2.0 | -52.2 | 5.1 | [S10] |
| C@$MoO_2$/G | 1.5 | -35.4 | 4.5 | [S11] |
| $Mo_2C$@C | 1.9 | -48.0 | 4.1 | [S12] |
| $Mo_2C$/C NCs | 2.6 | -49.2 | 4.6 | [S13] |
| $Mo_2N$@CoFe@C/CNT | 2.0 | -53.5 | 5.0 | This work |

## Supplementary References

[S1] J. Jiang, D. Li, D. Geng, J. An, J. He et al., Microwave absorption properties of core double-shell FeCo/C/$BaTiO_3$ nanocomposites. Nanoscale **6**, 3967-3971 (2014). https://doi.org/10.1039/C3NR04087A

[S2] Z. Yang, H. Lv, R. Wu, Rational construction of graphene oxide with mof-derived porous NiFe@C nanocubes for high-performance microwave attenuation. Nano Res. **9**, 3671-3682 (2016). https://doi.org/10.1007/s12274-

016-1238-z

[S3] B. Quan, X. Liang, G. Ji, J. Ma, P. Ouyang et al., Strong electromagnetic wave response derived from the construction of dielectric/magnetic media heterostructure and multiple interfaces. ACS Appl. Mater. Inter. **9**, 9964-9974 (2017). https://doi.org/10.1021/acsami.6b15788

[S4] Y. Wang, W. Zhang, X. Wu, C. Luo, Q. Wang et al., Conducting polymer coated metal-organic framework nanoparticles: Facile synthesis and enhanced electromagnetic absorption properties. Synthetic Met. **228**, 18-24 (2017). https://doi.org/10.1016/j.synthmet.2017.04.009

[S5] F. Wen, F. Zhang, Z. Liu, Investigation on microwave absorption properties for multiwalled carbon nanotubes/Fe/Co/Ni nanopowders as lightweight absorbers. J. Phys. Chem. C 115, 14025-14030 (2011). https://doi.org/10.1021/jp202078p

[S6] T. Wang, H. Wang, X. Chi, R. Li, J. Wang, Synthesis and microwave absorption properties of Fe-C nanofibers by electrospinning with disperse Fe nanoparticles parceled by carbon. Carbon **74**, 312-318 (2014). https://doi.org/10.1016/j.carbon.2014.03.037

[S7] R. Qiang, Y. Du, H. Zhao, Y. Wang, C. Tian et al., Metal organic framework-derived Fe/C nanocubes toward efficient microwave absorption. J. Mater. Chem. A **3**, 13426-13434 (2015). https://doi.org/10.1039/C5TA01457C

[S8] H. Liu, Y. Li, M. Yuan, G. Sun, H. Li et al., In situ preparation of cobalt nanoparticles decorated in n-doped carbon nanofibers as excellent electromagnetic wave absorbers. ACS Appl. Mater. Inter. **10**, 22591-22601 (2018). https://doi.org/10.1021/acsami.8b05211

[S9] X. Zhang, J. Xu, X. Liu, S. Zhang, H. Yuan et al., Metal organic framework-derived three-dimensional graphene-supported nitrogen-doped carbon nanotube spheres for electromagnetic wave absorption with ultralow filler mass loading. Carbon **155**, 233-242 (2019). https://doi.org/10.1016/j.carbon.2019.08.074

[S10] Z. Wu, K. Pei, L. Xing, X. Yu, W. You et al., Enhanced microwave absorption performance from magnetic coupling of magnetic nanoparticles suspended within hierarchically tubular composite. Adv. Funct. Mater. **29**, 1901448 (2019). https://doi.org/10.1002/adfm.201901448

[S11] C. Wu, Z. Chen, M. Wang, X. Cao, Y. Zhang et al., Confining tiny $MoO_2$ clusters into reduced graphene oxide for highly efficient low frequency microwave absorption. Small **16,** 2001686 (2020). https://doi.org/10.1002/smll.202001686

[S12] Y. Wang, X. Han, P. Xu, D. Liu, L. Cui et al., Synthesis of pomegranate-like $Mo_2C$@C nanospheres for highly efficient microwave absorption. Chem. Eng. J. **372**, 312-320 (2019). https://doi.org/10.1016/j.cej.2019.04.153

[S13] S. Dai, Y. Cheng, B. Quan, X. Liang, W. Liu et al., Porous-carbon-based $Mo_2C$ nanocomposites as excellent microwave absorber: A new exploration. Nanoscale **10**, 6945-6953 (2018). https://doi.org/10.1039/C8NR01244J
